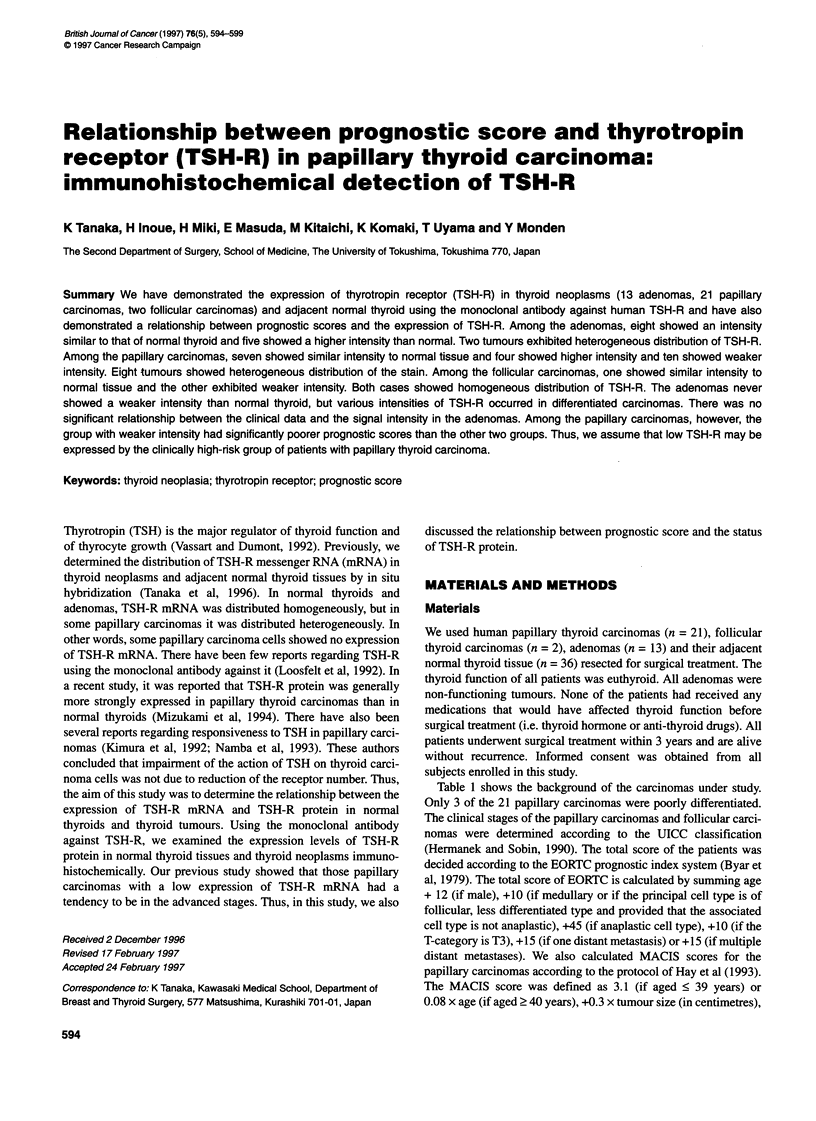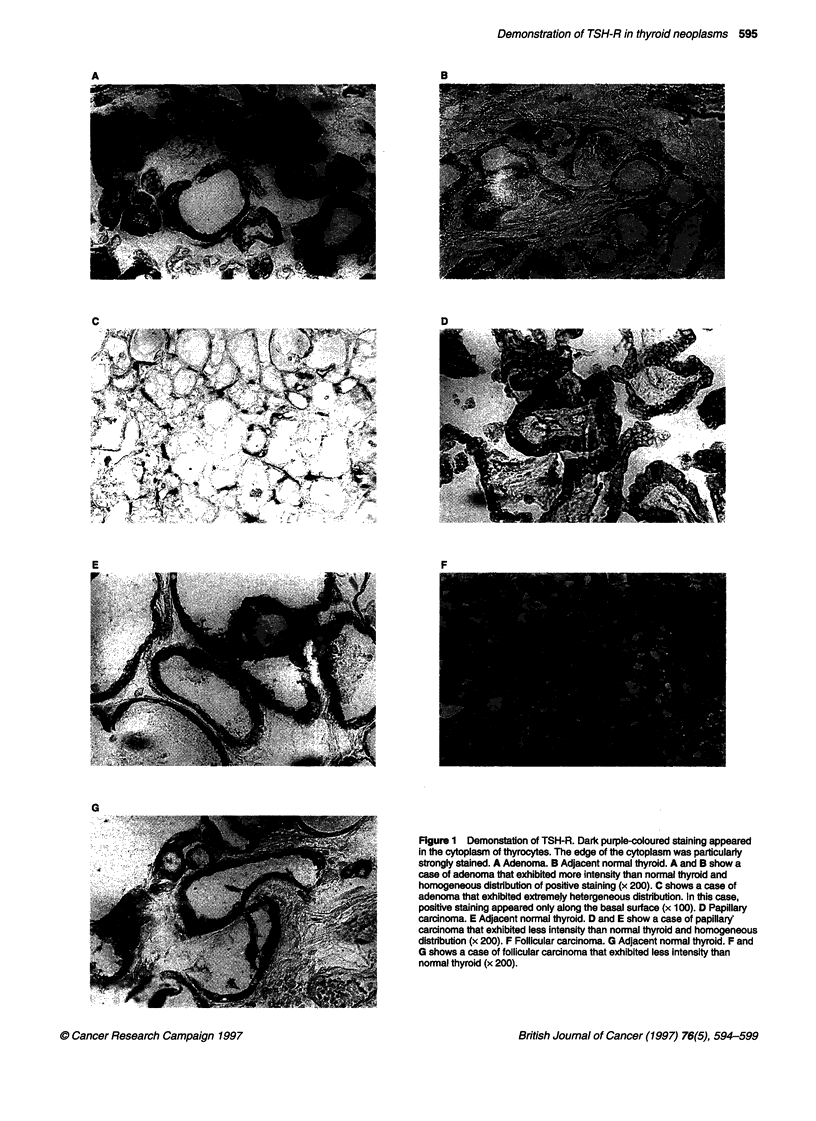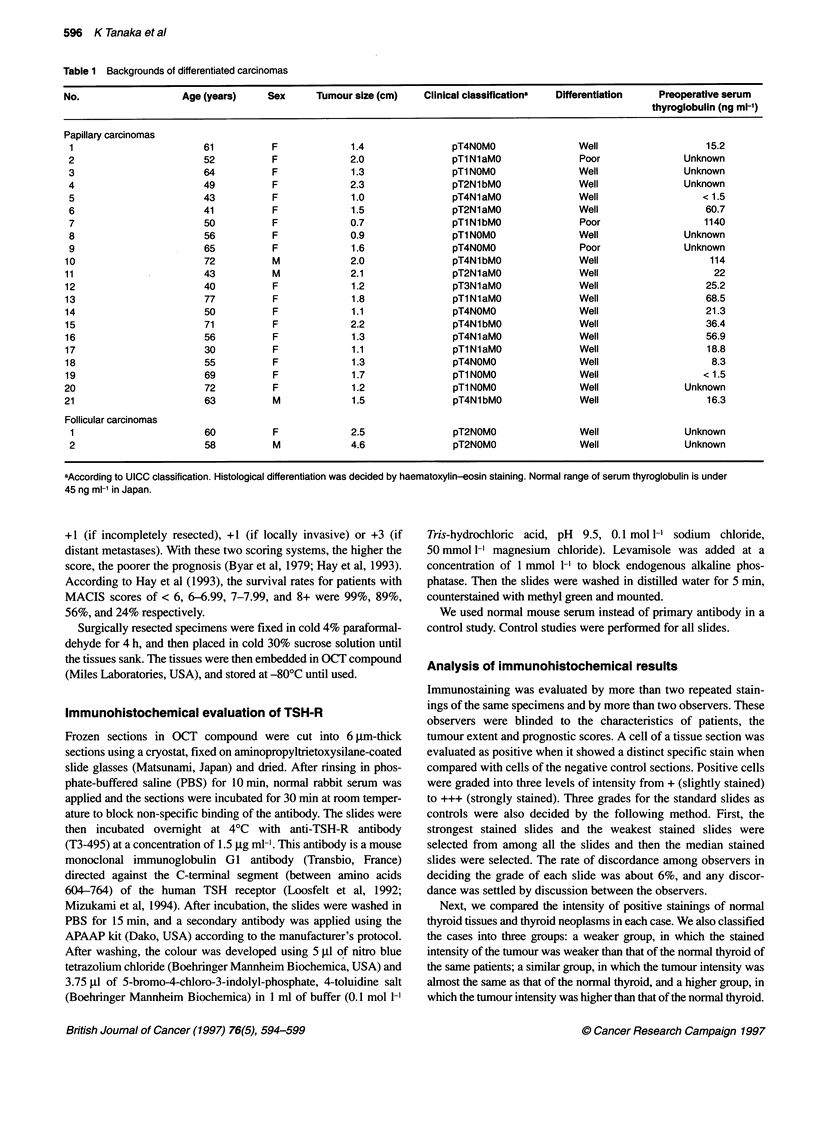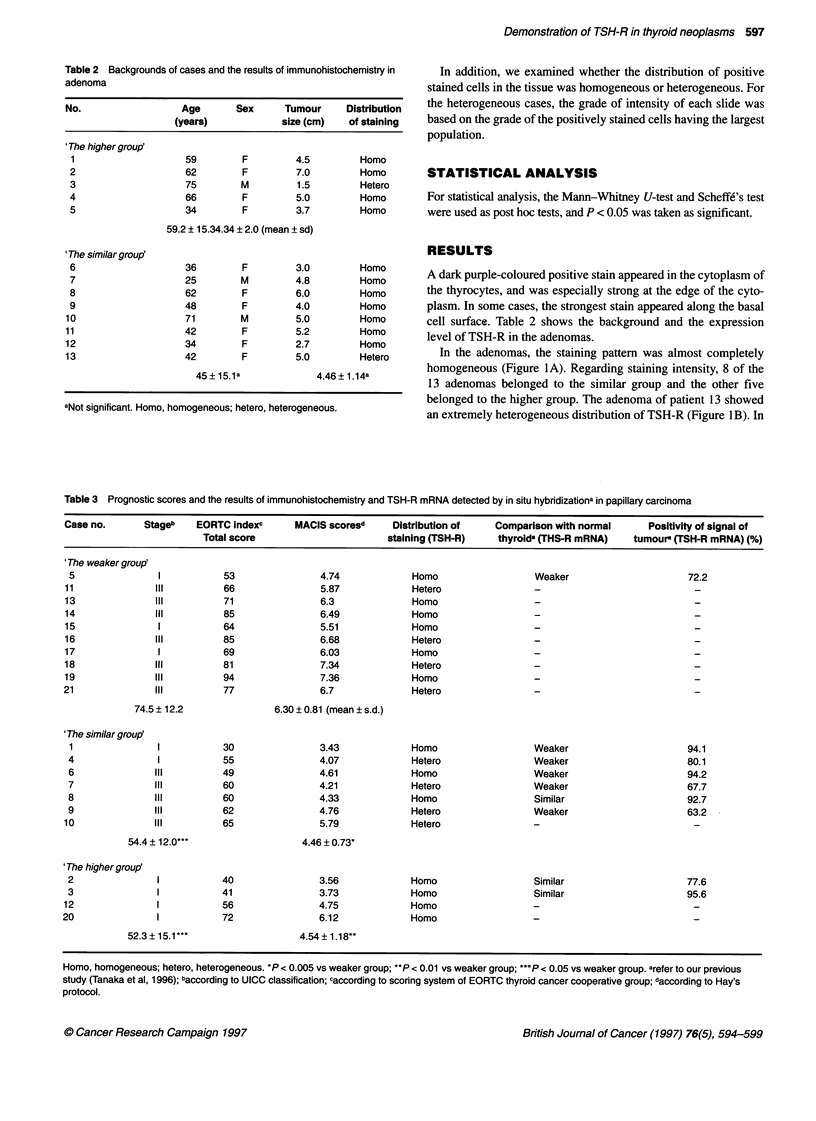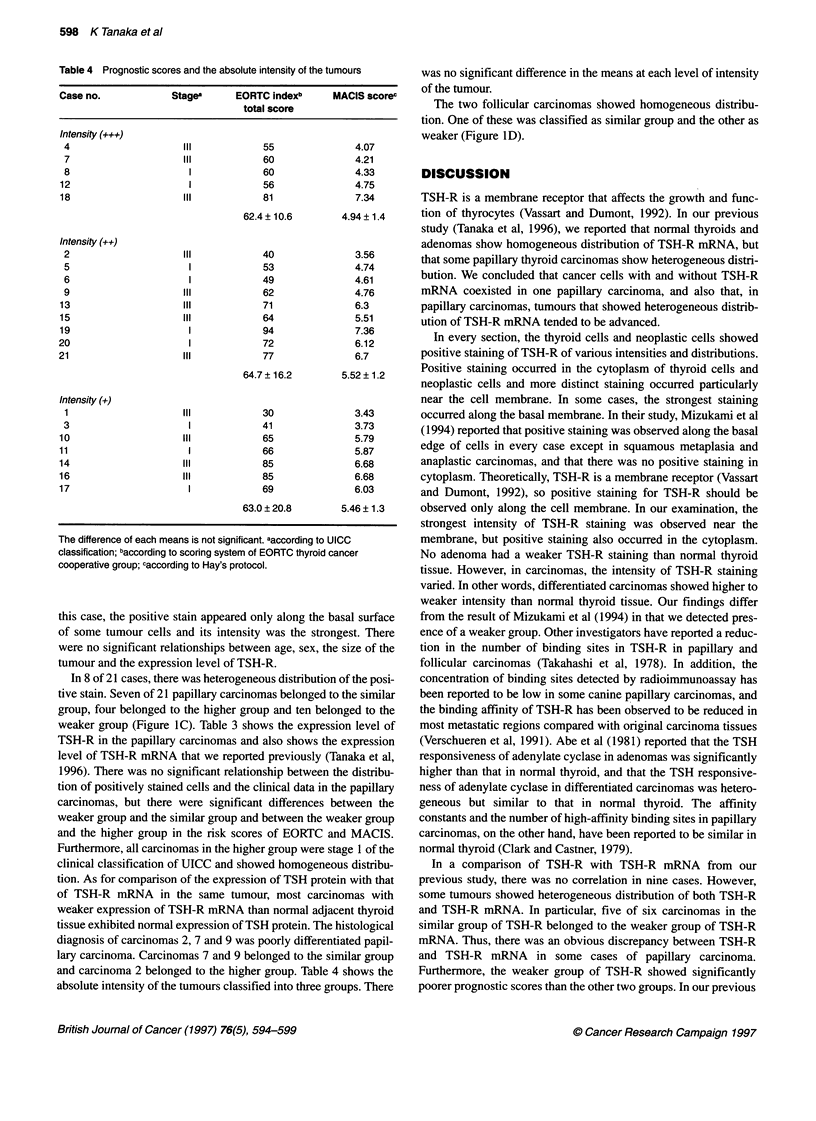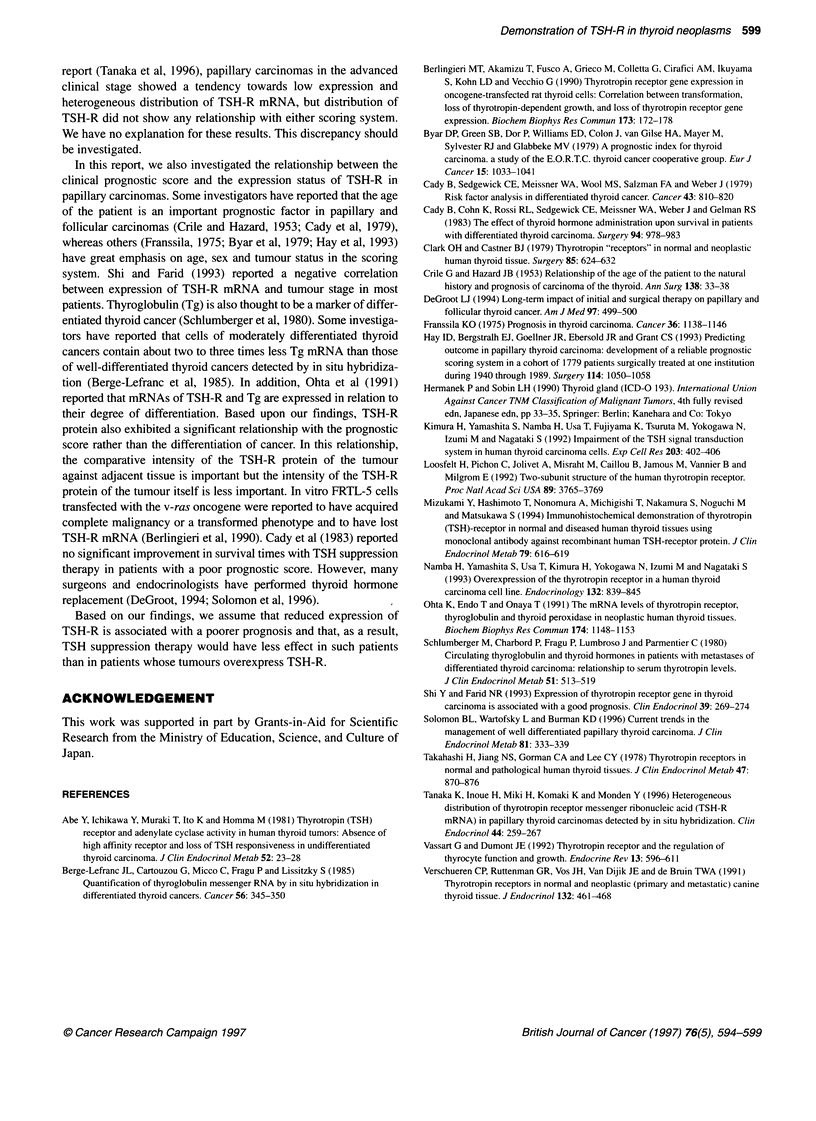# Relationship between prognostic score and thyrotropin receptor (TSH-R) in papillary thyroid carcinoma: immunohistochemical detection of TSH-R.

**DOI:** 10.1038/bjc.1997.431

**Published:** 1997

**Authors:** K. Tanaka, H. Inoue, H. Miki, E. Masuda, M. Kitaichi, K. Komaki, T. Uyama, Y. Monden

**Affiliations:** The Second Department of Surgery, School of Medicine, The University of Tokushima, Japan.

## Abstract

**Images:**


British Journal of Cancer (1997) 76(5), 594–599
© 1997 Cancer Research Campaign

### Relationship between prognostic score and thyrotropin receptor (TSH-R) in papillary thyroid carcinoma: immunohistochemical detection of TSH-R

**K Tanaka, H Inoue, H Miki, E Masuda, M Kitaichi, K Komaki, T Uyama and Y Monden**

The Second Department of Surgery, School of Medicine, The University of Tokushima, Tokushima 770, Japan

**Summary** We have demonstrated the expression of thyrotropin receptor (TSH-R) in thyroid neoplasms (13 adenomas, 21 papillary carcinomas, two follicular carcinomas) and adjacent normal thyroid using the monoclonal antibody against human TSH-R and have also demonstrated a relationship between prognostic scores and the expression of TSH-R. Among the adenomas, eight showed an intensity similar to that of normal thyroid and five showed a higher intensity than normal. Two tumours exhibited heterogeneous distribution of TSH-R. Among the papillary carcinomas, seven showed similar intensity to normal tissue and four showed higher intensity and ten showed weaker intensity. Eight tumours showed heterogeneous distribution of the stain. Among the follicular carcinomas, one showed similar intensity to normal tissue and the other exhibited weaker intensity. Both cases showed homogeneous distribution of TSH-R. The adenomas never showed a weaker intensity than normal thyroid, but various intensities of TSH-R occurred in differentiated carcinomas. There was no significant relationship between the clinical data and the signal intensity in the adenomas. Among the papillary carcinomas, however, the group with weaker intensity had significantly poorer prognostic scores than the other two groups. Thus, we assume that low TSH-R may be expressed by the clinically high-risk group of patients with papillary thyroid carcinoma.

**Keywords:** thyroid neoplasia; thyrotropin receptor; prognostic score

Thyrotropin (TSH) is the major regulator of thyroid function and of thyrocyte growth (Vassart and Dumont, 1992). Previously, we determined the distribution of TSH-R messenger RNA (mRNA) in thyroid neoplasms and adjacent normal thyroid tissues by in situ hybridization (Tanaka et al, 1996). In normal thyroids and adenomas, TSH-R mRNA was distributed homogeneously, but in some papillary carcinomas it was distributed heterogeneously. In other words, some papillary carcinoma cells showed no expression of TSH-R mRNA. There have been few reports regarding TSH-R using the monoclonal antibody against it (Loosfelt et al, 1992). In a recent study, it was reported that TSH-R protein was generally more strongly expressed in papillary thyroid carcinomas than in normal thyroids (Mizukami et al, 1994). There have also been several reports regarding responsiveness to TSH in papillary carcinomas (Kimura et al, 1992; Namba et al, 1993). These authors concluded that impairment of the action of TSH on thyroid carcinoma cells was not due to reduction of the receptor number. Thus, the aim of this study was to determine the relationship between the expression of TSH-R mRNA and TSH-R protein in normal thyroids and thyroid tumours. Using the monoclonal antibody against TSH-R, we examined the expression levels of TSH-R protein in normal thyroid tissues and thyroid neoplasms immunohistochemically. Our previous study showed that those papillary carcinomas with a low expression of TSH-R mRNA had a tendency to be in the advanced stages. Thus, in this study, we also discussed the relationship between prognostic score and the status of TSH-R protein.

*Received 2 December 1996*
*Revised 17 February 1997*
*Accepted 24 February 1997*

*Correspondence to:* K Tanaka, Kawasaki Medical School, Department of Breast and Thyroid Surgery, 577 Matsushima, Kurashiki 701-01, Japan

### MATERIALS AND METHODS

### Materials

We used human papillary thyroid carcinomas ($n$ = 21), follicular thyroid carcinomas ($n$ = 2), adenomas ($n$ = 13) and their adjacent normal thyroid tissue ($n$ = 36) resected for surgical treatment. The thyroid function of all patients was euthyroid. All adenomas were non-functioning tumours. None of the patients had received any medications that would have affected thyroid function before surgical treatment (i.e. thyroid hormone or anti-thyroid drugs). All patients underwent surgical treatment within 3 years and are alive without recurrence. Informed consent was obtained from all subjects enrolled in this study.

Table 1 shows the background of the carcinomas under study. Only 3 of the 21 papillary carcinomas were poorly differentiated. The clinical stages of the papillary carcinomas and follicular carcinomas were determined according to the UICC classification (Hermanek and Sobin, 1990). The total score of the patients was decided according to the EORTC prognostic index system (Byar et al, 1979). The total score of EORTC is calculated by summing age + 12 (if male), +10 (if medullary or if the principal cell type is of follicular, less differentiated type and provided that the associated cell type is not anaplastic), +45 (if anaplastic cell type), +10 (if the T-category is T3), +15 (if one distant metastasis) or +15 (if multiple distant metastases). We also calculated MACIS scores for the papillary carcinomas according to the protocol of Hay et al (1993). The MACIS score was defined as 3.1 (if aged ≤ 39 years) or 0.08 × age (if aged ≥ 40 years), +0.3 × tumour size (in centimetres),

**Figure 1** Demonstation of TSH-R. Dark purple-coloured staining appeared in the cytoplasm of thyrocytes. The edge of the cytoplasm was particularly strongly stained. **A** Adenoma. **B** Adjacent normal thyroid. **A** and **B** show a case of adenoma that exhibited more intensity than normal thyroid and homogeneous distribution of positive staining (× 200). **C** shows a case of adenoma that exhibited extremely hetergeneous distribution. In this case, positive staining appeared only along the basal surface (× 100). **D** Papillary carcinoma. **E** Adjacent normal thyroid. **D** and **E** show a case of papillary carcinoma that exhibited less intensity than normal thyroid and homogeneous distribution (× 200). **F** Follicular carcinoma. **G** Adjacent normal thyroid. **F** and **G** shows a case of follicular carcinoma that exhibited less intensity than normal thyroid (× 200).

*© Cancer Research Campaign 1997*

**Table 1** Backgrounds of differentiated carcinomas

| No. | Age (years) | Sex | Tumour size (cm) | Clinical classification[a] | Differentiation | Preoperative serum thyroglobulin (ng ml$^{-1}$) |
|---|---|---|---|---|---|---|
| Papillary carcinomas | | | | | | |
| 1 | 61 | F | 1.4 | pT4N0M0 | Well | 15.2 |
| 2 | 52 | F | 2.0 | pT1N1aM0 | Poor | Unknown |
| 3 | 64 | F | 1.3 | pT1N0M0 | Well | Unknown |
| 4 | 49 | F | 2.3 | pT2N1bM0 | Well | Unknown |
| 5 | 43 | F | 1.0 | pT4N1aM0 | Well | < 1.5 |
| 6 | 41 | F | 1.5 | pT2N1aM0 | Well | 60.7 |
| 7 | 50 | F | 0.7 | pT1N1bM0 | Poor | 1140 |
| 8 | 56 | F | 0.9 | pT1N0M0 | Well | Unknown |
| 9 | 65 | F | 1.6 | pT4N0M0 | Poor | Unknown |
| 10 | 72 | M | 2.0 | pT4N1bM0 | Well | 114 |
| 11 | 43 | M | 2.1 | pT2N1aM0 | Well | 22 |
| 12 | 40 | F | 1.2 | pT3N1aM0 | Well | 25.2 |
| 13 | 77 | F | 1.8 | pT1N1aM0 | Well | 68.5 |
| 14 | 50 | F | 1.1 | pT4N0M0 | Well | 21.3 |
| 15 | 71 | F | 2.2 | pT4N1bM0 | Well | 36.4 |
| 16 | 56 | F | 1.3 | pT4N1aM0 | Well | 56.9 |
| 17 | 30 | F | 1.1 | pT1N1aM0 | Well | 18.8 |
| 18 | 55 | F | 1.3 | pT4N0M0 | Well | 8.3 |
| 19 | 69 | F | 1.7 | pT1N0M0 | Well | < 1.5 |
| 20 | 72 | F | 1.2 | pT1N0M0 | Well | Unknown |
| 21 | 63 | M | 1.5 | pT4N1bM0 | Well | 16.3 |
| Follicular carcinomas | | | | | | |
| 1 | 60 | F | 2.5 | pT2N0M0 | Well | Unknown |
| 2 | 58 | M | 4.6 | pT2N0M0 | Well | Unknown |

[a]According to UICC classification. Histological differentiation was decided by haematoxylin–eosin staining. Normal range of serum thyroglobulin is under 45 ng ml$^{-1}$ in Japan.

+1 (if incompletely resected), +1 (if locally invasive) or +3 (if distant metastases). With these two scoring systems, the higher the score, the poorer the prognosis (Byar et al, 1979; Hay et al, 1993). According to Hay et al (1993), the survival rates for patients with MACIS scores of < 6, 6–6.99, 7–7.99, and 8+ were 99%, 89%, 56%, and 24% respectively.

Surgically resected specimens were fixed in cold 4% paraformaldehyde for 4 h, and then placed in cold 30% sucrose solution until the tissues sank. The tissues were then embedded in OCT compound (Miles Laboratories, USA), and stored at –80°C until used.

### Immunohistochemical evaluation of TSH-R

Frozen sections in OCT compound were cut into 6 μm-thick sections using a cryostat, fixed on aminopropyltrietoxysilane-coated slide glasses (Matsunami, Japan) and dried. After rinsing in phosphate-buffered saline (PBS) for 10 min, normal rabbit serum was applied and the sections were incubated for 30 min at room temperature to block non-specific binding of the antibody. The slides were then incubated overnight at 4°C with anti-TSH-R antibody (T3-495) at a concentration of 1.5 μg ml$^{-1}$. This antibody is a mouse monoclonal immunoglobulin G1 antibody (Transbio, France) directed against the C-terminal segment (between amino acids 604–764) of the human TSH receptor (Loosfelt et al, 1992; Mizukami et al, 1994). After incubation, the slides were washed in PBS for 15 min, and a secondary antibody was applied using the APAAP kit (Dako, USA) according to the manufacturer's protocol. After washing, the colour was developed using 5 μl of nitro blue tetrazolium chloride (Boehringer Mannheim Biochemica, USA) and 3.75 μl of 5-bromo-4-chloro-3-indolyl-phosphate, 4-toluidine salt (Boehringer Mannheim Biochemica) in 1 ml of buffer (0.1 mol l$^{-1}$ *Tris*-hydrochloric acid, pH 9.5, 0.1 mol l$^{-1}$ sodium chloride, 50 mmol l$^{-1}$ magnesium chloride). Levamisole was added at a concentration of 1 mmol l$^{-1}$ to block endogenous alkaline phosphatase. Then the slides were washed in distilled water for 5 min, counterstained with methyl green and mounted.

We used normal mouse serum instead of primary antibody in a control study. Control studies were performed for all slides.

### Analysis of immunohistochemical results

Immunostaining was evaluated by more than two repeated stainings of the same specimens and by more than two observers. These observers were blinded to the characteristics of patients, the tumour extent and prognostic scores. A cell of a tissue section was evaluated as positive when it showed a distinct specific stain when compared with cells of the negative control sections. Positive cells were graded into three levels of intensity from + (slightly stained) to +++ (strongly stained). Three grades for the standard slides as controls were also decided by the following method. First, the strongest stained slides and the weakest stained slides were selected from among all the slides and then the median stained slides were selected. The rate of discordance among observers in deciding the grade of each slide was about 6%, and any discordance was settled by discussion between the observers.

Next, we compared the intensity of positive stainings of normal thyroid tissues and thyroid neoplasms in each case. We also classified the cases into three groups: a weaker group, in which the stained intensity of the tumour was weaker than that of the normal thyroid of the same patients; a similar group, in which the tumour intensity was almost the same as that of the normal thyroid, and a higher group, in which the tumour intensity was higher than that of the normal thyroid.

© Cancer Research Campaign 1997

**Table 2** Backgrounds of cases and the results of immunohistochemistry in adenoma

| No. | Age (years) | Sex | Tumour size (cm) | Distribution of staining |
|---|---|---|---|---|
| *'The higher group'* | | | | |
| 1 | 59 | F | 4.5 | Homo |
| 2 | 62 | F | 7.0 | Homo |
| 3 | 75 | M | 1.5 | Hetero |
| 4 | 66 | F | 5.0 | Homo |
| 5 | 34 | F | 3.7 | Homo |
| | 59.2 ± 15.34.34 ± 2.0 (mean ± sd) | | | |
| *'The similar group'* | | | | |
| 6 | 36 | F | 3.0 | Homo |
| 7 | 25 | M | 4.8 | Homo |
| 8 | 62 | F | 6.0 | Homo |
| 9 | 48 | F | 4.0 | Homo |
| 10 | 71 | M | 5.0 | Homo |
| 11 | 42 | F | 5.2 | Homo |
| 12 | 34 | F | 2.7 | Homo |
| 13 | 42 | F | 5.0 | Hetero |
| | 45 ± 15.1[a] | | 4.46 ± 1.14[a] | |

[a]Not significant. Homo, homogeneous; hetero, heterogeneous.

In addition, we examined whether the distribution of positive stained cells in the tissue was homogeneous or heterogeneous. For the heterogeneous cases, the grade of intensity of each slide was based on the grade of the positively stained cells having the largest population.

### STATISTICAL ANALYSIS

For statistical analysis, the Mann–Whitney *U*-test and Scheffé's test were used as post hoc tests, and $P < 0.05$ was taken as significant.

### RESULTS

A dark purple-coloured positive stain appeared in the cytoplasm of the thyrocytes, and was especially strong at the edge of the cytoplasm. In some cases, the strongest stain appeared along the basal cell surface. Table 2 shows the background and the expression level of TSH-R in the adenomas.

In the adenomas, the staining pattern was almost completely homogeneous (Figure 1A). Regarding staining intensity, 8 of the 13 adenomas belonged to the similar group and the other five belonged to the higher group. The adenoma of patient 13 showed an extremely heterogeneous distribution of TSH-R (Figure 1B). In

**Table 3** Prognostic scores and the results of immunohistochemistry and TSH-R mRNA detected by in situ hybridization[a] in papillary carcinoma

| Case no. | Stage[b] | EORTC index[c] Total score | MACIS scores[d] | Distribution of staining (TSH-R) | Comparison with normal thyroid[a] (THS-R mRNA) | Positivity of signal of tumour[a] (TSH-R mRNA) (%) |
|---|---|---|---|---|---|---|
| *'The weaker group'* | | | | | | |
| 5 | I | 53 | 4.74 | Homo | Weaker | 72.2 |
| 11 | III | 66 | 5.87 | Hetero | – | – |
| 13 | III | 71 | 6.3 | Homo | – | – |
| 14 | III | 85 | 6.49 | Homo | – | – |
| 15 | I | 64 | 5.51 | Homo | – | – |
| 16 | III | 85 | 6.68 | Hetero | – | – |
| 17 | I | 69 | 6.03 | Homo | – | – |
| 18 | III | 81 | 7.34 | Hetero | – | – |
| 19 | III | 94 | 7.36 | Homo | – | – |
| 21 | III | 77 | 6.7 | Hetero | – | – |
| | 74.5 ± 12.2 | | 6.30 ± 0.81 (mean ± s.d.) | | | |
| *'The similar group'* | | | | | | |
| 1 | I | 30 | 3.43 | Homo | Weaker | 94.1 |
| 4 | I | 55 | 4.07 | Hetero | Weaker | 80.1 |
| 6 | III | 49 | 4.61 | Homo | Weaker | 94.2 |
| 7 | III | 60 | 4.21 | Hetero | Weaker | 67.7 |
| 8 | III | 60 | 4.33 | Homo | Similar | 92.7 |
| 9 | III | 62 | 4.76 | Hetero | Weaker | 63.2 |
| 10 | III | 65 | 5.79 | Hetero | – | – |
| | 54.4 ± 12.0*** | | 4.46 ± 0.73* | | | |
| *'The higher group'* | | | | | | |
| 2 | I | 40 | 3.56 | Homo | Similar | 77.6 |
| 3 | I | 41 | 3.73 | Homo | Similar | 95.6 |
| 12 | I | 56 | 4.75 | Homo | – | – |
| 20 | I | 72 | 6.12 | Homo | – | – |
| | 52.3 ± 15.1*** | | 4.54 ± 1.18** | | | |

Homo, homogeneous; hetero, heterogeneous. $^{*}P < 0.005$ vs weaker group; $^{**}P < 0.01$ vs weaker group; $^{***}P < 0.05$ vs weaker group. [a]refer to our previous study (Tanaka et al, 1996); [b]according to UICC classification; [c]according to scoring system of EORTC thyroid cancer cooperative group; [d]according to Hay's protocol.

© Cancer Research Campaign 1997

Table 4 Prognostic scores and the absolute intensity of the tumours

| Case no. | Stage[a] | EORTC index[b] total score | MACIS score[c] |
|---|---|---|---|
| *Intensity (+++)* | | | |
| 4 | III | 55 | 4.07 |
| 7 | III | 60 | 4.21 |
| 8 | I | 60 | 4.33 |
| 12 | I | 56 | 4.75 |
| 18 | III | 81 | 7.34 |
| | | 62.4 ± 10.6 | 4.94 ± 1.4 |
| *Intensity (++)* | | | |
| 2 | III | 40 | 3.56 |
| 5 | I | 53 | 4.74 |
| 6 | I | 49 | 4.61 |
| 9 | III | 62 | 4.76 |
| 13 | III | 71 | 6.3 |
| 15 | III | 64 | 5.51 |
| 19 | I | 94 | 7.36 |
| 20 | I | 72 | 6.12 |
| 21 | III | 77 | 6.7 |
| | | 64.7 ± 16.2 | 5.52 ± 1.2 |
| *Intensity (+)* | | | |
| 1 | III | 30 | 3.43 |
| 3 | I | 41 | 3.73 |
| 10 | III | 65 | 5.79 |
| 11 | I | 66 | 5.87 |
| 14 | III | 85 | 6.68 |
| 16 | III | 85 | 6.68 |
| 17 | I | 69 | 6.03 |
| | | 63.0 ± 20.8 | 5.46 ± 1.3 |

The difference of each means is not significant. [a]according to UICC classification; [b]according to scoring system of EORTC thyroid cancer cooperative group; [c]according to Hay's protocol.

this case, the positive stain appeared only along the basal surface of some tumour cells and its intensity was the strongest. There were no significant relationships between age, sex, the size of the tumour and the expression level of TSH-R.

In 8 of 21 cases, there was heterogeneous distribution of the positive stain. Seven of 21 papillary carcinomas belonged to the similar group, four belonged to the higher group and ten belonged to the weaker group (Figure 1C). Table 3 shows the expression level of TSH-R in the papillary carcinomas and also shows the expression level of TSH-R mRNA that we reported previously (Tanaka et al, 1996). There was no significant relationship between the distribution of positively stained cells and the clinical data in the papillary carcinomas, but there were significant differences between the weaker group and the similar group and between the weaker group and the higher group in the risk scores of EORTC and MACIS. Furthermore, all carcinomas in the higher group were stage 1 of the clinical classification of UICC and showed homogeneous distribution. As for comparison of the expression of TSH protein with that of TSH-R mRNA in the same tumour, most carcinomas with weaker expression of TSH-R mRNA than normal adjacent thyroid tissue exhibited normal expression of TSH protein. The histological diagnosis of carcinomas 2, 7 and 9 was poorly differentiated papillary carcinoma. Carcinomas 7 and 9 belonged to the similar group and carcinoma 2 belonged to the higher group. Table 4 shows the absolute intensity of the tumours classified into three groups. There was no significant difference in the means at each level of intensity of the tumour.

The two follicular carcinomas showed homogeneous distribution. One of these was classified as similar group and the other as weaker (Figure 1D).

### DISCUSSION

TSH-R is a membrane receptor that affects the growth and function of thyrocytes (Vassart and Dumont, 1992). In our previous study (Tanaka et al, 1996), we reported that normal thyroids and adenomas show homogeneous distribution of TSH-R mRNA, but that some papillary thyroid carcinomas show heterogeneous distribution. We concluded that cancer cells with and without TSH-R mRNA coexisted in one papillary carcinoma, and also that, in papillary carcinomas, tumours that showed heterogeneous distribution of TSH-R mRNA tended to be advanced.

In every section, the thyroid cells and neoplastic cells showed positive staining of TSH-R of various intensities and distributions. Positive staining occurred in the cytoplasm of thyroid cells and neoplastic cells and more distinct staining occurred particularly near the cell membrane. In some cases, the strongest staining occurred along the basal membrane. In their study, Mizukami et al (1994) reported that positive staining was observed along the basal edge of cells in every case except in squamous metaplasia and anaplastic carcinomas, and that there was no positive staining in cytoplasm. Theoretically, TSH-R is a membrane receptor (Vassart and Dumont, 1992), so positive staining for TSH-R should be observed only along the cell membrane. In our examination, the strongest intensity of TSH-R staining was observed near the membrane, but positive staining also occurred in the cytoplasm. No adenoma had a weaker TSH-R staining than normal thyroid tissue. However, in carcinomas, the intensity of TSH-R staining varied. In other words, differentiated carcinomas showed higher to weaker intensity than normal thyroid tissue. Our findings differ from the result of Mizukami et al (1994) in that we detected presence of a weaker group. Other investigators have reported a reduction in the number of binding sites in TSH-R in papillary and follicular carcinomas (Takahashi et al, 1978). In addition, the concentration of binding sites detected by radioimmunoassay has been reported to be low in some canine papillary carcinomas, and the binding affinity of TSH-R has been observed to be reduced in most metastatic regions compared with original carcinoma tissues (Verschueren et al, 1991). Abe et al (1981) reported that the TSH responsiveness of adenylate cyclase in adenomas was significantly higher than that in normal thyroid, and that the TSH responsiveness of adenylate cyclase in differentiated carcinomas was heterogeneous but similar to that in normal thyroid. The affinity constants and the number of high-affinity binding sites in papillary carcinomas, on the other hand, have been reported to be similar in normal thyroid (Clark and Castner, 1979).

In a comparison of TSH-R with TSH-R mRNA from our previous study, there was no correlation in nine cases. However, some tumours showed heterogeneous distribution of both TSH-R and TSH-R mRNA. In particular, five of six carcinomas in the similar group of TSH-R belonged to the weaker group of TSH-R mRNA. Thus, there was an obvious discrepancy between TSH-R and TSH-R mRNA in some cases of papillary carcinoma. Furthermore, the weaker group of TSH-R showed significantly poorer prognostic scores than the other two groups. In our previous

© Cancer Research Campaign 1997

report (Tanaka et al, 1996), papillary carcinomas in the advanced clinical stage showed a tendency towards low expression and heterogeneous distribution of TSH-R mRNA, but distribution of TSH-R did not show any relationship with either scoring system. We have no explanation for these results. This discrepancy should be investigated.

In this report, we also investigated the relationship between the clinical prognostic score and the expression status of TSH-R in papillary carcinomas. Some investigators have reported that the age of the patient is an important prognostic factor in papillary and follicular carcinomas (Crile and Hazard, 1953; Cady et al, 1979), whereas others (Franssila, 1975; Byar et al, 1979; Hay et al, 1993) have great emphasis on age, sex and tumour status in the scoring system. Shi and Farid (1993) reported a negative correlation between expression of TSH-R mRNA and tumour stage in most patients. Thyroglobulin (Tg) is also thought to be a marker of differentiated thyroid cancer (Schlumberger et al, 1980). Some investigators have reported that cells of moderately differentiated thyroid cancers contain about two to three times less Tg mRNA than those of well-differentiated thyroid cancers detected by in situ hybridization (Berge-Lefranc et al, 1985). In addition, Ohta et al (1991) reported that mRNAs of TSH-R and Tg are expressed in relation to their degree of differentiation. Based upon our findings, TSH-R protein also exhibited a significant relationship with the prognostic score rather than the differentiation of cancer. In this relationship, the comparative intensity of the TSH-R protein of the tumour against adjacent tissue is important but the intensity of the TSH-R protein of the tumour itself is less important. In vitro FRTL-5 cells transfected with the v-*ras* oncogene were reported to have acquired complete malignancy or a transformed phenotype and to have lost TSH-R mRNA (Berlingieri et al, 1990). Cady et al (1983) reported no significant improvement in survival times with TSH suppression therapy in patients with a poor prognostic score. However, many surgeons and endocrinologists have performed thyroid hormone replacement (DeGroot, 1994; Solomon et al, 1996).

Based on our findings, we assume that reduced expression of TSH-R is associated with a poorer prognosis and that, as a result, TSH suppression therapy would have less effect in such patients than in patients whose tumours overexpress TSH-R.

### ACKNOWLEDGEMENT

This work was supported in part by Grants-in-Aid for Scientific Research from the Ministry of Education, Science, and Culture of Japan.

### REFERENCES

Abe Y, Ichikawa Y, Muraki T, Ito K and Homma M (1981) Thyrotropin (TSH) receptor and adenylate cyclase activity in human thyroid tumors: Absence of high affinity receptor and loss of TSH responsiveness in undifferentiated thyroid carcinoma. *J Clin Endocrinol Metab* **52**: 23–28

Berge-Lefranc JL, Cartouzou G, Micco C, Fragu P and Lissitzky S (1985) Quantification of thyroglobulin messenger RNA by in situ hybridization in differentiated thyroid cancers. *Cancer* **56**: 345–350

Berlingieri MT, Akamizu T, Fusco A, Grieco M, Colletta G, Cirafici AM, Ikuyama S, Kohn LD and Vecchio G (1990) Thyrotropin receptor gene expression in oncogene-transfected rat thyroid cells: Correlation between transformation, loss of thyrotropin-dependent growth, and loss of thyrotropin receptor gene expression. *Biochem Biophys Res Commun* **173**: 172–178

Byar DP, Green SB, Dor P, Williams ED, Colon J, van Gilse HA, Mayer M, Sylvester RJ and Glabbeke MV (1979) A prognostic index for thyroid carcinoma. a study of the E.O.R.T.C. thyroid cancer cooperative group. *Eur J Cancer* **15**: 1033–1041

Cady B, Sedgewick CE, Meissner WA, Wool MS, Salzman FA and Weber J (1979) Risk factor analysis in differentiated thyroid cancer. *Cancer* **43**: 810–820

Cady B, Cohn K, Rossi RL, Sedgewick CE, Meissner WA, Weber J and Gelman RS (1983) The effect of thyroid hormone administration upon survival in patients with differentiated thyroid carcinoma. *Surgery* **94**: 978–983

Clark OH and Castner BJ (1979) Thyrotropin "receptors" in normal and neoplastic human thyroid tissue. *Surgery* **85**: 624–632

Crile G and Hazard JB (1953) Relationship of the age of the patient to the natural history and prognosis of carcinoma of the thyroid. *Ann Surg* **138**: 33–38

DeGroot LJ (1994) Long-term impact of initial and surgical therapy on papillary and follicular thyroid cancer. *Am J Med* **97**: 499–500

Franssila KO (1975) Prognosis in thyroid carcinoma. *Cancer* **36**: 1138–1146

Hay ID, Bergstralh EJ, Goellner JR, Ebersold JR and Grant CS (1993) Predicting outcome in papillary thyroid carcinoma: development of a reliable prognostic scoring system in a cohort of 1779 patients surgically treated at one institution during 1940 through 1989. *Surgery* **114**: 1050–1058

Hermanek P and Sobin LH (1990) Thyroid gland (ICD-O 193). *International Union Against Cancer TNM Classification of Malignant Tumors*, 4th fully revised edn, Japanese edn, pp 33–35, Springer: Berlin; Kanehara and Co: Tokyo

Kimura H, Yamashita S, Namba H, Usa T, Fujiyama K, Tsuruta M, Yokogawa N, Izumi M and Nagataki S (1992) Impairment of the TSH signal transduction system in human thyroid carcinoma cells. *Exp Cell Res* **203**: 402–406

Loosfelt H, Pichon C, Jolivet A, Misraht M, Caillou B, Jamous M, Vannier B and Milgrom E (1992) Two-subunit structure of the human thyrotropin receptor. *Proc Natl Acad Sci USA* **89**: 3765–3769

Mizukami Y, Hashimoto T, Nonomura A, Michigishi T, Nakamura S, Noguchi M and Matsukawa S (1994) Immunohistochemical demonstration of thyrotropin (TSH)-receptor in normal and diseased human thyroid tissues using monoclonal antibody against recombinant human TSH-receptor protein. *J Clin Endocrinol Metab* **79**: 616–619

Namba H, Yamashita S, Usa T, Kimura H, Yokogawa N, Izumi M and Nagataki S (1993) Overexpression of the thyrotropin receptor in a human thyroid carcinoma cell line. *Endocrinology* **132**: 839–845

Ohta K, Endo T and Onaya T (1991) The mRNA levels of thyrotropin receptor, thyroglobulin and thyroid peroxidase in neoplastic human thyroid tissues. *Biochem Biophys Res Commun* **174**: 1148–1153

Schlumberger M, Charbord P, Fragu P, Lumbroso J and Parmentier C (1980) Circulating thyroglobulin and thyroid hormones in patients with metastases of differentiated thyroid carcinoma: relationship to serum thyrotropin levels. *J Clin Endocrinol Metab* **51**: 513–519

Shi Y and Farid NR (1993) Expression of thyrotropin receptor gene in thyroid carcinoma is associated with a good prognosis. *Clin Endocrinol* **39**: 269–274

Solomon BL, Wartofsky L and Burman KD (1996) Current trends in the management of well differentiated papillary thyroid carcinoma. *J Clin Endocrinol Metab* **81**: 333–339

Takahashi H, Jiang NS, Gorman CA and Lee CY (1978) Thyrotropin receptors in normal and pathological human thyroid tissues. *J Clin Endocrinol Metab* **47**: 870–876

Tanaka K, Inoue H, Miki H, Komaki K and Monden Y (1996) Heterogeneous distribution of thyrotropin receptor messenger ribonucleic acid (TSH-R mRNA) in papillary thyroid carcinomas detected by in situ hybridization. *Clin Endocrinol* **44**: 259–267

Vassart G and Dumont JE (1992) Thyrotropin receptor and the regulation of thyrocyte function and growth. *Endocrine Rev* **13**: 596–611

Verschueren CP, Ruttenman GR, Vos JH, Van Dijik JE and de Bruin TWA (1991) Thyrotropin receptors in normal and neoplastic (primary and metastatic) canine thyroid tissue. *J Endocrinol* **132**: 461–468

© Cancer Research Campaign 1997